# Cracking the whip: spatial voting with party discipline and voter polarization

**DOI:** 10.1007/s11127-017-0463-7

**Published:** 2017-06-26

**Authors:** T. D. P. Waters

**Affiliations:** Institute for Fiscal Studies, London, UK

**Keywords:** Spatial voting, Hotelling, Whipping, Legislative discipline, Polarization, Elections, D72

## Abstract

The spatial voting theory literature has generally focused on either parties or candidates as the unit of analysis and ignored strategic interactions between them. I study a game theoretic spatial model of elections with many heterogeneous constituencies in which both party and candidate behavior are modeled. Parties choose a platform and a ‘whip rate,’ representing the proportion of final policy that will be made by the party, as opposed to by the successful candidates. Candidates are office-motivated and can choose both a platform and a level of advertising in order to defeat their opponent. It is shown that the introduction of whipping as a choice variable can cause party platforms to diverge and that parties will whip on some but not all issues, reflecting the empirical reality of parties influencing rather than determining policy outcomes exclusively. Further, parties respond to sharper voter polarization by reducing the power of the whip as well as distinguishing their platforms from one another, while more voter uncertainty has the opposite effect. Other real-world phenomena, including ‘safe seats’ and legislators voting with their party even when unwhipped, are also shown to be predicted by the model.

## Introduction

In the classic Hotelling–Downs model (Hotelling 1929; Downs 1957) of elections, two parties or candidates who care only about winning the election compete over a spectrum of voters in a single district, and equilibrium emerges when both position themselves at the median voter’s location. In that model, and many others in the field, the party is a single unit and the winner decides policy unilaterally. Other papers model many constituencies that elect one legislator each (i.e., single-member districts), and policy is purely a function of candidates’ choices. Instead, in this paper I model parties and candidates as separate strategic actors. I introduce ‘whipping’ into the spatial voting framework, and it is partly through whipping that the relationship between a candidate and her party is mediated. Parties are independent decision makers who, as well as choosing party platforms, whip their candidates to follow the party line on certain issues, but allow ‘free votes’ on others. The existence of whipping means that voters know that if they elect a candidate of a given party, the agenda of that party is more likely to be successful. This means that the party’s platform, as well as the candidate’s platform, is relevant to their decision making. Whipping has not been introduced previously in the spatial voting literature, and only one other paper models candidates and parties as separate strategic actors.

I answer three questions: Firstly, does the existence of whipping as a choice variable for parties result in party platform differentiation, rather than the median voter result? Secondly, how does voter polarization affect the frequency with which parties whip and the platforms they choose? Thirdly, how realistic a picture of electoral politics does a model with whipping and voter polarization provide?

I find that the existence of whipping as a choice variable does result in platform differentiation under many parameterizations and that parties will choose to whip some of the agenda but allow legislators freedom elsewhere. However, the classic Downsian median voter result can occur under other parameterizations. I find that sharper voter polarization tends to increase party platform differentiation and reduce the proportion of policy that is whipped. I also show that voters’ uncertainty about candidate platforms affects whipping and platform divergence. Finally, I argue in the conclusion that, in a number of respects, this model predicts electoral phenomena which are mirrored in the real world: not only party platform divergence and parties whipping some but not all policy, but also the existence of both ‘safe’ seats to which incumbents are reelected with certainty and other seats to which they are not, and legislators who tend to vote somewhat in line with their party’s ideology even in free votes.

The paper is structured as follows. In Sect. 2, I discuss the relevant literature. In Sect. 3, I set out the model. In Sect. 4, I find what strategies voters and candidates will play. That tells us what equilibrium candidate utility will be as a function of party choices, as well as the probability that any given candidate is elected. In Sect. 5, I use the results found for equilibrium candidate utility to simulate party choices and find how the equilibria react to a change in voter polarization or uncertainty. Further, I indicate how legislators from each party will vote in free votes, to show that even when unwhipped, they are likely to vote in a way reflective of their party’s ideology. I also test how the results change when parties aim to win seats for their own sake. Finally, I conclude in Sect. 6.

## Relationship to the literature

Much of the literature on spatial voting has focused on models assuming a single election and a single constituency of voters. That is unlike many real-world elections, such as elections to the US Congress, which are composed of many small races. An extant literature models behavior under those sorts of systems with multiple constituencies. We can split these papers into three groups according to who they model as strategic players: only parties, only candidates, or both.

An early paper by Austen-Smith (1981) modeled parties as the relevant decision makers. As well as choosing political platforms, parties also have monetary endowments. The funds can be spent on a ‘deposit’ to allow a candidate to stand in a particular constituency, and, as in the model I propose, the money can also be allocated to political advertising. If parties aim to maximize seats, Austen-Smith finds that an equilibrium exists, and that so long as parties have different monetary endowments, they will also differ in platform choices.

Callander (2005) extended an earlier model by Palfrey (1984). In the Palfrey model, there is a single constituency with two ‘national’ parties, and elections are two-stage games. In the first stage, the national parties choose their platforms and then an independent party, having observed the national party positions, enters the race and chooses a platform. The result is that national parties platforms do diverge; however, the third party always loses the election, raising the question of why they compete in the first place. Callander improves on this model by introducing many heterogeneous constituencies. The national parties have to choose the same platform in every constituency, but an independent can run in only one of them. It turns out that as long as constituencies are not too polarized, the national parties will diverge in such a way that no independent can enter the race and have any chance of winning.

A third paper modeling only the behavior of parties is Castanheira and Crutzen (2006). That paper has some similarities with the model I develop. Parties are given two choice variables: political platform and a screening technology, which the authors term ‘discipline.’ If discipline is strong, the range within which candidates are randomly drawn is narrowed; that is, candidates are more likely to have ideological positions close to the party’s platform. Voters are not informed of the candidates’ ideological leanings, but do know the parties’ platforms and degree of discipline, and so know the probability distribution of candidate platforms. They find that parties will discipline candidates fully, so that all candidates will have the same ideological position as the party. The variable of ‘discipline’ is rather different from ‘whipping’ in my model, as it functions as a method of screening which candidates are chosen, rather than restricting the behavior of candidates once they are in the legislature. A related study is that by Merrill et al. (2014). They suppose that parties might have an exogenous ‘tether,’ similar in principle to Castanheira and Crutzen’s screening device, which limits the extent to which candidates can diverge from party platforms. Party platforms for the next election are the average of winning candidate platforms for the previous one. Parties may wish to tether, or discipline, candidates in this way in order to maintain the party brand, or to pass their legislative agenda. The authors show how party platform divergence varies under different types and strictnesses of tethers, which can be thought of as a measure of party discipline. As party and candidate behavior is not modeled, the paper does not represent an *explanation* of the extent of party discipline, but the mechanism proposed in the paper suggests that discipline will be positively correlated with party platform divergence. The present essay (while using a different definition of discipline) suggests the reverse result.

Other papers have candidates as the only actors in the model. A pair of papers by Austen-Smith (1984, 1987) modeled each candidate choosing a platform, and then the party policy is generated by the ‘party constitution,’ which is a function of candidate platforms. In the second paper, constitutions are allowed to take several forms. Perhaps the most interesting form occurs when the platform of every candidate contributes to the party constitution. Voters know the constitutions and the platforms of all candidates, and so know what party policy will be. Austen-Smith finds that the conditions required for a coalition-proof equilibrium is quite restrictive on the party constitution. However, if the constitutions abide by these conditions, the overall position of each party must be the same; there is no divergence in party platforms.

Another pair of papers, like the Austen-Smith ones, model candidate behavior with party platform as a function of candidate choices. Snyder (1994) proposes a system in which only incumbent legislators get to vote on their party’s platform for the next election. Legislators want to keep their seats, and so those representing a left-wing constituency will naturally want their party’s policy to be left wing. He finds that party platforms diverge from one another so that every incumbent legislator is always reelected. However, the divergence in platforms that the model permits is extremely small.

The second paper in this vein, Ansolabehere et al. (2012), takes the same basic principle of letting incumbent legislators decide the next election’s policy, but adds in random shocks, representing something like a ‘scandal,’ which parties cannot influence, but affects voter choices. Because of the risk of a scandal causing a legislator to lose her seat, incumbents have stronger preferences over party policy; in the Snyder (1994) paper, so long as the constituency’s median voter slightly prefers party *x*’s policy, the *x* candidate would win with certainty. In this model with shocks, it is always strictly better for a candidate to have her party policy move closer to her constituency’s median voter. The result of this is that party platforms may diverge sharply.

Finally, Eyster and Kittsteiner (2007) model both candidates and parties as independent agents. It appears that, aside from the present essay, this is the only extant paper that allows both types of agents to be decision makers. In their system, parties choose platforms in order to maximize the aggregate utility of the party’s candidates. Candidates select their own platforms, and the winning candidate alone implements policy for her constituency. However, candidates have to incur a cost if they differ from their own party’s platform, increasing in the distance between platforms. The authors find that, for certain calibrations of parameters, party platforms diverge, in order to save candidates from paying too large a cost.

## The model

The political world is populated by many constituencies. Each constituency is comprised of a continuum of voters. These constituencies may differ in the views of local voters.

Two political parties, *A* and *B*, are assumed. I use capital letters to refer to parties, and lower case letters for candidates. Both parties field one candidate each in every constituency. No other candidates run for office. In each constituency, the candidate with the majority of votes wins the seat.

There are *R* policy issues on which parties, candidates and voters have platforms or preferences. For each of those issues, there is a unidimensional and continuous ideological space. Issues also have a ‘weight,’ or salience parameter $\rho _{r}$ for issue *r*, and $\sum _{r=1}^{R}\rho _{r}=1$. These parameters are the same across voters (in other words, all voters see a given issue as having the same level of importance, even if they have different preferences with respect to that issue). Voters have ‘ideological bliss points’ in the ideological space for each issue. To maintain the tractability of the model, I assume that a given voter’s ideological bliss points are the same across all policy issues (so a left-wing voter is left wing on all policies). However, I do not impose that restriction on parties or candidates, who may announce left-wing platforms on some issues and right wing platforms on others, although, as I will show later, they find it optimal to announce the same position on all issues.

The ideological bliss point of the median voter in a given constituency will be of interest later, and I bound the median voter’s bliss point to be between zero and one. Hence, constituencies with a median voter who has a bliss point near 0 can be thought of as ‘left-wing’ constituencies, whereas constituencies with a median voter who has a bliss point near 1 are ‘right wing.’ Define *g*(*y*) as the density of constituencies with a median voter with a bliss point of *y*. *G*(*y*) is the corresponding CDF. I assume that *G*(*y*) is continuous and strictly increasing in *y*.

Both parties and candidates choose platforms for policy issues. Parties also choose which issues they will ‘whip,’ i.e., require their legislators to adhere to the party line. For issues on which they whip, parties announce platforms. For those issues that parties do not whip, their candidates announce platforms independently. Candidates also choose how much advertising they will do to inform the public of their platforms, denoted $e_{a}$ and $e_{b}$ for candidates *a* and *b*, respectively.

These platforms and whip choices affect final policy outcomes in the following way. Once the election has occurred, the winning party (the one with a majority of seats) implements its platform on the issues that they whip by whipping their successful legislative candidates to support the party’s position. I assume that the penalty for failing to abide by the whip is sufficiently strong to dissuade any legislator from rebelling. Thus, the party platform can be thought of as a manifesto: a set of policies that will pass if the party wins. However, not all policies are determined by the manifesto. The unwhipped issues are decided by ‘free votes,’ where legislators are allowed to ‘vote their consciences.’ This is where candidates’ platforms come in: in free votes, successful candidates vote according to their personal platforms.

Voters evaluate party and candidate platforms in the following way. For each issue *r*, they receive disutility in proportion to the distance between their ideological bliss point and the platform of either the candidate or the party (according to whether or not *r* is whipped). Without loss of generality, suppose that party *A* whips issues $1,\ldots ,{\bar{r}}$, and so has a platform on those issues only and candidate *a* therefore has a platform on issues ${\bar{r}}+1,\ldots ,R$. Denote party *A*’s platform on issue *r* as $\theta _{A,r}$, the *A* party candidate’s platform in constituency *i* on issue *s* as $\theta _{a,i,s}$, and the ideological bliss point of voter *j* (who is in *i*’s constituency) as $y_{j}$. Then the utility that *j* would receive if candidate $a_{i}$ was elected is:

$$\begin{aligned} u_{j}(a)= -\left( \sum _{r=1}^{{\bar{r}}}\rho _{r}|\theta _{A,r}-y_{j}| +\sum _{s={\bar{r}}+1}^{R}\rho _{s}|\theta _{a,i,s}-y_{j}|\right) \end{aligned}$$

The utility received from candidate $b_{i}$ being elected is equivalent. Voter *j* will vote for candidate *a* if $u_{j}(a)>u_{j}(b)$. If the inequality is reversed, then voter *j* votes for *b*; with an equality, *j* votes for each candidate with probability one-half.

This decision rule assumes that voters are ‘sincere’; that is, they vote for the policy that, if implemented, would be closest to their ideological bliss points. In this way, they do not take into account the behavior of other voters, particularly voters in different constituencies. As Ortuno-Ortin explains:

An agent votes for the policy that, were it implemented, would give him a higher utility level. So voters only compare the two proposals and not the possible final outcomes. Thus, agents are assumed to be less sophisticated than parties. This is a common restriction in this area of research.... A possible justification for it is that there is a continuum of agents. In this case a single vote cannot affect the implemented policy. (Ortuno-Ortin 1997)

Voters know with certainty the position of each party on the ideological spectra of issues, but treat the candidates’ platforms as random variables. This assumption reflects the reality that, while it is comparatively easy to know the political positions of national parties, whose policies are well publicized, it is much harder to know what one’s local candidates believe. Indeed, a 2013 survey found that only 22% of UK voters could name their local Member of Parliament (Hansard Society 2013). The variable denoting candidate *a*’s perceived platform in constituency *i* on issue *s* is:

$$\begin{aligned} {\bar{\theta }}_{a,i,s}\sim U\left( \theta _{a,i,s}-2\left( \sigma -e_{a,i}\right) ,\theta _{a,i,s}+2\left( \sigma -e_{a,i}\right) \right) ,\quad {\hbox{where U is the uniform distribution}} \end{aligned}$$

Candidate *a* chooses $\theta _{a,i,s}$ (his or her ‘true’ platform) and $e_{a,i}$ (the amount of advertising they do to publicize their platform, which reduces voter uncertainty). Parameter $\sigma$ is a measure of the uncertainty about the candidate’s position if no advertising is done. Clearly $e_{a,i}\le \sigma$.

Candidates are motivated only by winning office, but face a cost of advertising, *c*(*e*), where $c(\cdot )$ is continuously differentiable, $c^{\prime }(e)>0$ and $c(0)=0$. The value to a candidate of winning the election is $V>0$. This gives a utility function for candidates of party *A* in constituency *i* of:

$$\begin{aligned} v_{a,i}={\left\{ \begin{array}{ll} V-c(e_{a,i}) & {\hbox{if a wins}}\\ -c(e_{a,i}) & {\hbox{if b wins}} \end{array}\right. } \end{aligned}$$

Following Eyster and Kittsteiner (2007), I begin with the assumption that parties aim to maximize their candidates’ total welfare; party utility is the sum of candidates’ utilities. This assumption will be relaxed later.

The timing is as follows:

1. The issues that parties will whip and the associated party platforms ($\{\theta _{A,1},\ldots ,\theta _{A,{\bar{r}}}\},\{\theta _{B,1},\ldots ,\theta _{B,{\bar{r}}}\}$) are chosen simultaneously.
2. Candidates observe both parties’ platforms and which issues are whipped, and all candidates choose their own platforms and advertising effort simultaneously.
3. The election takes place.

## Constituency equilibrium

In order to determine what choices parties will make, we need to know what happens at the constituency level, given the choices of parties. For the sake of brevity I generally will give definitions and strategies from the ‘perspective’ of candidate or party *A*, though symmetric statements are true for *B*. Before proceeding to examine how voters will vote, it is convenient to first note that:

### **Proposition 1**

*Parties’ and candidates’ optimal platform choices always include choosing the same position for all issues rather than different positions for different issues; that is, if party* *A* *whips issues* $1,\ldots ,{\bar{r}}$, *they will find that setting* $\theta _{A,r}=\theta _{A}\forall r\in \{1,\ldots ,{\bar{r}}\}$, *for some platform* $\theta _{A}$ *will be optimal; similarly, setting* $\theta _{a,i,s}=\theta _{a,i}\forall s\in \{{\bar{r}}+1,\ldots ,R\}$ *for some platform* $\theta _{a,i}$ *will be optimal for* *a*.

### *Proof*

See “Appendix”.□

I assume that parties and candidates follow this optimal strategy. Note that generically it will not be the case that $\theta _{A}=\theta _{a,i}$. From now on I therefore will simply refer to a party or candidate platform in the singular. A definition is helpful:

### **Definition 1**

If party *A* whips issues $1,\ldots ,{\bar{r}}$, then *A*’s whip rate is $w_{A}=\sum _{r=1}^{{\bar{r}}}\rho _{r}$.

This is the proportion of issues that the party whips, weighted by their salience.

### Voter strategies

For simplicity I drop constituency subscripts at this point, and so refer to candidate *a*’s platform $\theta _{a}$ and his or her advertising effort $e_{a}$, rather than $\theta _{a,i}$ and $e_{a,i}$.

Proposition 1 and Definition 1 allow us to simplify the utility of voter *j* from *a* winning to:

$$\begin{aligned} u_{j}(a)=&-\left( w_{A}|\theta _{A}-y_{j}|+(1-w_{A})|{\bar{\theta }}_{a}-y_{j}|\right) \end{aligned}$$

The reader will notice that this rendering of $u_{j}$ does not refer directly to separate issues and their saliences, as the relevant aspects of the party and candidate strategies can be summarized by the whip rate and uniform party and candidate platforms. I assume that voters in a given constituency are sufficiently homogeneous in their ideologies that the ‘median voter’ result within a constituency prevails. That is, if the median voter votes for candidate *a*, then candidate *a* wins the election. This is a strong assumption, because we cannot guarantee that preferences are single-peaked, and so at least half of the voters might have to be in a reasonably tight space to generate the median voter result. However, it allows us to focus our attention entirely on the median voter’s expected utility, and who she votes for.

### Candidate strategies

As stated previously, party *A*’s utility is the sum of the utilities of all of *A*’s candidates. Hence, we need to find candidate utility as a function of party choices (whip rates and platforms) and the constituency median. Further, the probability that any given candidate is elected also will be useful; I use this later to work out how successful candidates from each party vote in ‘unwhipped’ free votes, after they enter the legislature.

In order to answer both of these questions, we need to know what strategy candidates will play.

By the time candidates are making their decisions, $\theta _{A}$, $\theta _{B}$, $w_{A}$, and $w_{B}$ are known. Letting *m* denote the position of the median voter in the constituency and $u_{m}(a)$ her utility if *a* is elected, taking expectations over ${\bar{\theta }}_{a}$ gives:

$$\begin{aligned} E[u_{m}(a)]={\left\{ \begin{array}{ll}-\left( w_{A}|\theta _{A}-m|+(1-w_{A})(m-\theta _{a})\right) & \quad \text{ for } 2(\sigma -e_{a})\le m-\theta _{a}\\ -\left( w_{A}|\theta _{A}-m|+(1-w_{A})\left( \frac{\left( \theta _{a}-m\right) ^{2}+(2(\sigma -e_{a}))^{2}}{4(\sigma -e_{a})}\right) \right) & \quad \text{ for } -2(\sigma -e_{a})<m-\theta _{a}<2(\sigma -e_{a})\\ -\left( w_{A}|\theta _{A}-m|+(1-w_{A})(\theta _{a}-m)\right) & \quad \text{ for } m-\theta _{a}\le -2(\sigma -e_{a})\end{array}\right. } \end{aligned}$$

It is relatively straightforward to see that it is weakly dominant for candidate *a* to play $\theta _{a}=m$ and for candidate *b* to play $\theta _{b}=m$. I assume that candidates will set $\theta _{a}=\theta _{b}=m$ from now on. The platform choices for candidates are then quite trivial. The interest comes in the level of advertising they choose, and this is what I examine for most of the rest of this section.

Given these platforms, the expected utility of the median voter from *a* being elected is:

4.1 $$\begin{aligned} {\mathbb {E}}[u_{m}(a)]=-\left( w_{A}|\theta _{A}-m| +(1-w_{A})(\sigma -e_{a})\right) \end{aligned}$$

#### **Definition 2**

$H(m):=w_{B}(|\theta _{B}-m|-\sigma )-w_{A}(|\theta _{A}-m|-\sigma )$ is candidate *a*’s headstart in a constituency with median *m*.

This is a ‘headstart’ for *a* in the sense that if neither candidate advertises, it is the extra utility that the median voter receives if she votes for candidate *a* rather than *b*. The headstart function will be referred to repeatedly below, and in this section stated simply as *H*. It is worth re-emphasizing that the candidates take *H* as given, as well as $w_{A}$ and $w_{B}$.

Candidate *a* wins the election if:

$$\begin{aligned} H>&e_{b}(1-w_{B})-e_{a}(1-w_{A}) \end{aligned}$$

This result comes from equation (4.1) and its equivalent for ${\mathbb {E}}[u_{m}(b)]$. Similarly, *b* wins if the inequality is reversed, and each wins with probability $\frac {1}{2}$ if it is an equality.

Before finding the candidates’ equilibrium strategies, we need a further assumption:

#### **Assumption 1**

$$\sigma \ge c^{-1}(V)$$

This is necessary because:

#### **Proposition 2**

*If* $\sigma <c^{-1}(V)$, *a Nash equilibrium does not generally exist*

#### *Proof*

See “Appendix”.□

I now move on to finding equilibrium strategies. Clearly, no candidate will set advertising above $c^{-1}(V).$ If a candidate did so, utility would be negative even if he or she won, as the cost of advertising would exceed the benefit of winning. Hence, playing $e_{i}>c^{-1}(V)$ is strictly dominated by $e_{i}=0$.

#### **Definition 3**

An election is uncontested in *a*’s favor if $H\ge c^{-1}(V)(1-w_{B})$

If an election is uncontested in *a*’s favor, the implication is that even if *b* expended the maximum amount of advertising possible ($c^{-1}(V)$) and *a* spent nothing, the race would either be a tie or *a* would win. These are ‘uncontested’ in the sense that neither candidate has an incentive to do any advertising at all, because the headstart of one candidate is so large.

#### **Definition 4**

Candidate *a* is lower advantaged if $H>0$.

#### **Definition 5**

Candidate *a* is upper advantaged if $H>c^{-1}(V)(w_{A}-w_{B})$

The intuition behind these definitions is as follows. A candidate is lower advantaged if, when neither advertizes ($e_{a}=e_{b}=0$), that candidate wins. A candidate is upper advantaged if, when both do the maximum amount of advertising ($e_{a}=e_{b}=c^{-1}(V)$), that candidate wins. It is possible that a candidate has both, one, or neither type of advantage.

We can now move onto finding equilibrium in constituencies.

#### **Lemma 1**

1. *If* *a* *is lower advantaged*, *then for* *b*, *all* $e_{b}\in \left( 0,\frac{H}{1-w_{B}}\right)$ *are strictly dominated.*
2. *If* *a* *is upper advantaged (or neither candidate is upper advantaged), then for* *a*, *all* $e_{a}>\frac{c^{-1}(V)(1-w_{B})-H}{1-w_{A}}$ *are strictly dominated.*

#### *Proof*

See “Appendix”.□

Define the largest $e_{a}$ that *a* is prepared to play as ${\bar{e_{a}}}$, so

$$\begin{aligned} {\bar{e_{a}}}={\left\{ \begin{array}{ll} \frac{c^{-1}(V)(1-w_{B})-H}{1-w_{A}} & \quad {\hbox{if a is upper advantaged}}\\ c^{-1}(V) & \quad {\hbox{if b is upper advantaged}} \end{array}\right. } \end{aligned}$$

We define $\bar{e_{b}}$ similarly. It will also be useful to define, for the lower *dis*advantaged candidate only, $\underline{e}_{i}$ for $i=a,b$ as the smallest $e_{i}$ that is not strictly dominated *excluding* zero. So, if *a* is lower advantaged, $\underline{e}_{b}:=\frac{H}{1-w_{B}}$; if *b* is lower advantaged, $\underline{e}_{a}:=\frac{-H}{1-w_{A}}$.

Define a ‘mass point’ in a strategy as a level of $e_{i}$ that a candidate plays with positive probability. Then:

#### **Lemma 2**

*Neither player puts a mass point on any* $e_{i}$ *other than zero.*

#### *Proof*

See “Appendix”.□

A ‘hole’ in a strategy is defined in the following way. There is a hole between *x* and *y* ($x<y$) in *i*’s strategy if and only if $F_{i}(x)=F_{i}(y)$, where $F_{i}$ describes the CDF of *i*’s advertising probability distribution. Then:

#### **Lemma 3**

*In contested elections:*

1. *If a candidate with lower advantage has a hole in her strategy between* *y* *and* *z* (with $y<z$), *then she has a hole between* *y* *and* ${\bar{e}}_{i}$.
2. *If a candidate without lower advantage has a hole between* *y* *and* *z* (*with* $y<z$), *then she has a hole between* *y* *and* ${\bar{e}}_{i}$, *unless* $0<y<\underline{e}_{i}$.

#### *Proof*

See “Appendix”.□

So far, we know that the candidate with lower advantage may put mass on zero and nowhere else and then may mix continuously over a range from zero to some $y\le {\bar{e}}_{i}$. The candidate without lower advantage may put mass on zero and nowhere else and then may mix continuously over a range from $\underline{e}_{i}$ to some $z\le {\bar{e}}_{i}$.

#### **Lemma 4**

*Suppose that at least one candidate is either lower or upper advantaged. Then:*

1. *If one candidate does not have lower advantage and the other does not have upper advantage, the first does not put mass on zero but the second does.* 1
2. *If one candidate is upper and lower advantaged, both candidates put mass on zero.*

#### *Proof*

See “Appendix”.□

Lemma 4 provides us with another helpful property of constituency equilibria. The candidate who is not upper advantaged always puts mass on zero, but always loses when she plays zero. Suppose that *a* is upper advantaged and lower disadvantaged. Then, from part 1 of Lemma 4, *a* does not play zero, so always plays at least $\underline{e}_{a}$—which is enough to beat *b* when *b* plays zero. If *a* is upper and lower advantaged, then if *b* plays zero she loses whatever *a* does. So this means that the upper *dis*advantaged candidate—in this case *b*—has an equilibrium payoff of zero. This result gives us:

#### **Lemma 5**

*If the election is contested, both candidates have* ${\bar{e}}_{i}$ *in their support*

#### *Proof*

See “Appendix”.□

This, combined with Lemma 3 then implies that the lower advantaged candidate must mix across $(0,{\bar{e}}_{i})$, and the lower disadvantaged candidate must mix across $(\underline{e}_{i},{\bar{e}}_{i})$, without holes in both cases.

To sum up what we have learned from these lemmata:

#### **Proposition 3**

*If the election is contested and at least one candidate has at least one type of advantage:*

*If* *a* *is upper and lower advantaged, then she will place mass on *0 *only, and mix continuously across* $(0,\frac{c^{-1}(V)(1-w_{B})-H}{1-w_{A}})$; *while* *b* *will place mass on *0 *only, and mix continuously across* $( \frac{H}{1-w_{B}},c^{-1}(V))$.

*If* *a* *is not lower advantaged and* *b* *is not upper advantaged, then* *a* *will place mass nowhere, and will mix continuously across* $(\frac{-H}{1-w_{A}},\frac{c^{-1}(V)(1-w_{B})-H}{1-w_{A}})$; *while* *b* *will place mass on *0 *only, and mix continuously across* $(0,c^{-1}(V))$.

With these results in hand, we can now turn to characterizing the equilibrium. I describe the equilibrium in terms of the optimal strategy for candidates *a* and *b*, $F_{a}^{*}(e_{a})$ and $F_{b}^{*}(e_{b})$. These are the CDFs of advertising costs.

#### **Theorem 1**

*Given* $w_{A}$, $w_{B}$, *A*, *B* *and* *m*, *the candidates’ equilibrium strategies are:*

E1.: *If the election is uncontested, then neither candidate advertises.*
E2.: *If candidate* *a* *is not lower advantaged and* *b* *is not* *upper advantaged* 2 *:*
  $$\begin{aligned} F_{a}^{*}(x)&={\left\{ \begin{array}{ll} 0 & \text {for }\,x<\frac{-H}{1-w_{A}}\\ \frac{1}{V}c\Biggl (\frac{x(1-w_{A})+H}{1-w_{B}}\Biggr ) & \text {for }\,\frac{-H}{1-w_{A}}\le x<\frac{c^{-1}(V)(1-w_{B})-H}{1-w_{A}}\\ 1 & \text {for }\,\frac{c^{-1}(V)(1-w_{B})-H}{1-w_{A}}\le x \end{array}\right. }\\ F_{b}^{*}(x)&= {\left\{ \begin{array}{ll} 0 & \text {for }\,x<0\\ 1-\frac{1}{V}\Biggl (c\Biggl (\frac{c^{-1}(V)(1-w_{B})-H)}{1-w_{A}}\Biggr )-c\Biggl (\frac{x(1-w_{B})-H)}{1-w_{A}}\Biggr )\Biggr ) & \text {for }\,0\le x\le c^{-1}(V)\\ 1 & \text {for }\,c^{-1}(V)<x \end{array}\right. } \end{aligned}$$
E3.: *If candidate* *a* *is both upper and lower advantaged, then:*
  $$\begin{aligned} F_{a}^{*}(x)=&{\left\{ \begin{array}{ll} 0 & \quad \text {for }x<0\\ \frac{1}{V}c\left( \frac{x(1-w_{A})+H}{1-w_{B}}\right) & \quad {\text {for }\,0\le x\le \frac{c^{-1}(V)(1-w_{B})-H}{1-w_{A}}}\\ 1 & \quad \text {for }\,\frac{c^{-1}(V)(1-w_{B})-H}{1-w_{A}}<x \end{array}\right. }\\ F_{b}^{*}(x)=&{\left\{ \begin{array}{ll} 0 & \quad \text {for }x<0\\ 1-\frac{1}{V}c\left( \frac{c^{-1}(V)(1-w_{B})-H)}{1-w_{A}}\right) & \quad \text {for }\,0\le x\le \frac{H}{1-w_{B}}\\ 1-\frac{1}{V}\left( c\left( \frac{c^{-1}(V)(1-w_{B})-H)}{1-w_{A}}\right) -c\left( \frac{x(1-w_{B})-H)}{1-w_{A}}\right) \right) & \quad \text {for }\,\frac{H}{1-w_{B}}<x\le c^{-1}(V)\\ 1 & \quad \text {for }\,c^{-1}(V)<x \end{array}\right. } \end{aligned}$$

#### *Proof*

See “Appendix”.□

Symmetric equilibria exist for E2 and E3 where *b* has the advantage. Having determined equilibrium strategies, we can now state the equilibrium utility for candidates:

#### **Proposition 4**

*If the election is uncontested in candidate* *a*’*s favor alone, then* ${\mathbb {E}}[v_{a}]=V$, ${\mathbb {E}}[v_{b}]=0$. *If* *a* *has upper advantage, then* ${\mathbb {E}}[v_{a}]=V-c\left( \frac{c^{-1}(V)(1-w_{B})-H}{1-w_{A}}\right)$, ${\mathbb {E}}[v_{b}]=0$.

Symmetric payoffs exist if *b* has an uncontested win or upper advantage. Note that which player has lower advantage does not affect utility: for the rest of this paper lower advantage is no longer a particularly important concept.

This proposition gives candidate utility as a function of the constituency median and party choices. Hence in Sect. 5 we can derive party utility (the sum of their candidates’ utilities) as a function of party choices.

One special case merits discussion. It is possible for the election to be uncontested in *both* *a* and *b*’s favor. This happens when $H=c^{-1}(V)(1-w_{B})=-c^{-1}(V)(1-w_{A})$, which implies that $w_{A}=w_{B}=1$ (and that party platforms are equidistant from the median voter). In this case, neither candidate advertises at all (since it does not affect their chances of winning), and they both win with probability $\frac {1}{2}$. Then, ${\mathbb {E}}[v_{a}]={\mathbb {E}}[v_{b}]=\frac {V}{2}$.

Before moving on to look at the overall party equilibrium, I derive the probability of a candidate winning their constituency election. This will be useful later in looking at how successful candidates behave in free votes, after they have entered the legislature. Firstly, define $\gamma :=\frac{c^{-1}(V)(1-w_{B})-H}{1-w_{A}}$. Then:

#### **Theorem 2**

*If the equilibrium is E1, then the candidate in whose favor the election is uncontested will win with certainty, unless the election is uncontested in the favor of both candidates, in which case each wins with probability* $\frac {1}{2}$.

*If the equilibrium is E2:*

$$\begin{aligned} \Pr (a\text { wins})=&\int _{\frac{-H}{1-w_{A}}}^{\gamma }\left[ 1-\frac{1}{V}\left( c(\gamma )-c(x)\right) \right] \frac{1}{V}c^{\prime }\left( \frac{x(1-w_{A})+H}{1-w_{B}}\right) \left( \frac{1-w_{A}}{1-w_{B}}\right) \,{\text {d}}x \end{aligned}$$

*If the equilibrium is E3:*

$$\begin{aligned} \Pr (a\,\text {wins})= & \left( 1-\frac{1}{V}c(\gamma )\right) \frac{1}{V}c\left( \frac{H}{1-w_{B}}\right) \\&+\,\int _{0}^{\gamma }\left[ 1-\frac{1}{V}\left( c(\gamma )-c(x)\right) \right] \frac{1}{V}c^{\prime }\left( \frac{x(1-w_{A})+H}{1-w_{B}}\right) \left( \frac{1-w_{A}}{1-w_{B}}\right) {\text {d}}x \end{aligned}$$

#### *Proof*

See “Appendix”.□

## Party equilibrium

In this section, I solve for party choices in equilibrium and find how they respond to changing parameters. I also give an indication of how legislators (successful candidates) vote in free votes.

Parties aim to maximize the aggregate utility of their candidates. From the last section, we now know what any given candidate’s utility is, as a function of party platforms, whip rates and the constituency median. Recall that the constituencies are heterogeneous, in the sense that constituency medians vary. Because candidates receive utility only when the election is uncontested in their favor or when they are upper advantaged (see Proposition 4), parties also receive utility only in constituencies where that is the case.

**Fig. 1:**
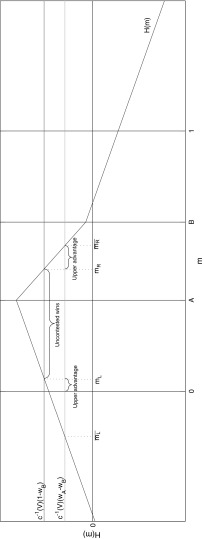
Example *H*(*m*), with $$w_{A}=0.7$$, $$w_{B}=0.3$$, $$\theta _{A}=0.35$$, $$\theta _{B}=0.65$$, $$\sigma =0.6$$, $$c^{-1}(V)=0.3$$

To build intuition, it is helpful to look at the shape of an example headstart function in Fig. 1.

Recall that *A* wins uncontested elections when $H(m)\ge c^{-1}(V)(1-w_{B})$ and is upper advantaged when $H(m)>c^{-1}(V)(w_{A}-w_{B})$. In Fig. 1, $m_{L}^{*}$ and $m_{R}^{*}$ are the ‘cut-points’ that identify the space over which *A* wins uncontested elections; similarly $\bar{m_{L}}$ and $\bar{m_{R}}$ are the cut-points that identify when *A* has upper advantage. In this case, both $m_{L}^{*}$ and $m_{R}^{*}$ are in [0, 1]. However, the same is not true for the upper advantage cut-points. The left one is less than zero, but no constituencies exist with medians less than zero. Hence, the space over which *A* has upper advantage is between zero and $m_{L}^{*}$ and between $m_{R}^{*}$ and $\bar{m_{R}}$.

To solve for the Nash equilibrium, I simulate the game.^3^ In order to calculate the equilibria, we need to give numerical values to exogenous parameters. Two functions—$c(\cdot )$ and $G(\cdot )$—and two scalar parameters—*V*, $\sigma$—are in play.

For $c(\cdot )$, I set $c(e)=\frac{e}{\alpha }$. This means that costs are linear, and marginal cost is constant. Linear costs have the virtue of being simple, requiring only a single new parameter ($\alpha$). It has the disadvantage that it is implausible insofar as it implies that candidates can reduce uncertainty about themselves with constant marginal returns. That seems unlikely. There are quite cheap ways to educate the public to a reasonable degree about a candidate’s position by using ‘short cuts.’ For example, the candidate could state, ‘I am center-left’ or ‘I broadly agree with my party’s manifesto,’ which provides quite a lot of information at low cost. However, once voters already are relatively certain about a candidate’s position, fewer short cuts will be available and so advertising will require specific policy statements, which is more costly as each issue has to be addressed separately. Nonetheless, the simplicity of the linear cost model is an important property, and so I use it for simulation.

For $G(\cdot )$, I use a (discretized) normal distribution with a mean of $\frac {1}{2}$. The variance is a measure of the polarization of voters. The greater the variance of $G(\cdot )$, the more constituencies exist with relatively extreme median voters. This allows us to model the relationship between voter polarization and both party discipline and platform divergence.

To find the equilibrium for a given set of parameters, I uniformly discretize the platform space, constituency space and whip rates into 101 grid points from 0 to 1. I then find strategies where *A* and *B* are mutually best replying.

*V* and $\alpha$ work in almost exactly opposite directions: a larger value from winning the election is very much like a lower cost of advertising. Owing to space constraints, I do not show how the equilibrium changes in response to changes in *V* and $\alpha$.^4^

Three types of result emerge from the model: ‘Downsian’ equilibrium, divergent platform equilibrium and no pure Nash equilibrium exists.^5^

An equilibrium is ‘Downsian’ if both parties set their whip rates to 1 and their platforms to $\frac {1}{2}$. Here, parties are homogeneous units (candidates do not have their own platforms) and parties position themselves at the median constituency, mimicking the classic Downsian result. A divergent platform equilibrium arises when parties play different platforms, but the same whip rate.^6^ Platforms vary around the median constituency, so one party is at $\frac {1}{2}-x$ and the other at $\frac {1}{2}+x$ $\exists x>0$. It appears that no pure Nash equilibrium exists when voter polarization is very large or voter uncertainty is very small.^7^ Further research could identify what mixed equilibria are available at these parameterizations.

**Fig. 2:**
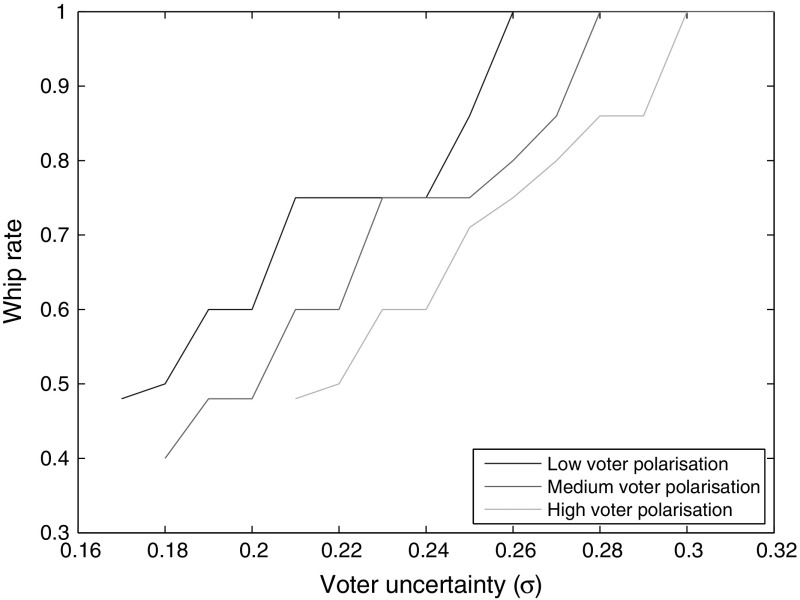
Equilibrium whip rate as a function of $\sigma$, for different variances of *G* (0.20, 0.24, 0.28)

**Fig. 3:**
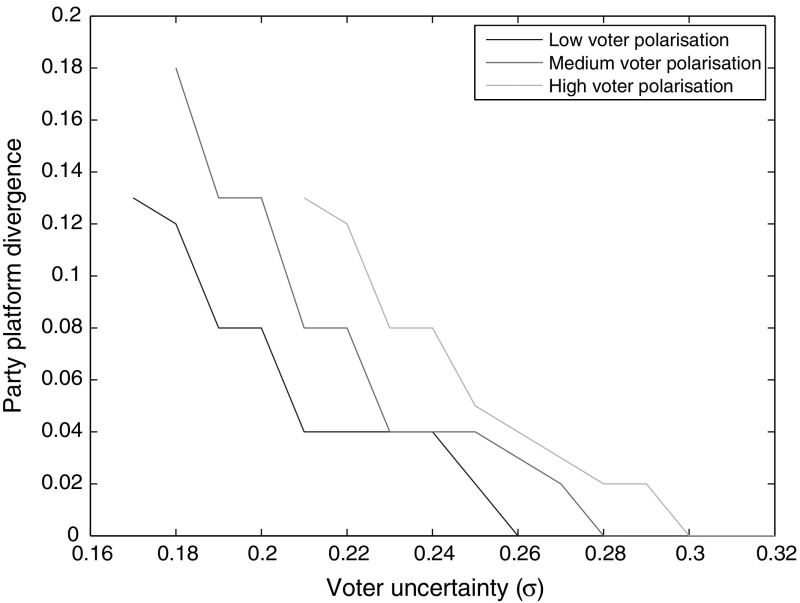
Equilibrium platform divergence as a function of $\sigma$, for different variances of *G* (0.20, 0.24, 0.28)

The effect of a change in voter uncertainty on platform divergence and whip rate is affected heavily by the level of headstart (*H*(*m*)) that must be reached for a constituency to be uncontested in a party’s favor. For *A*, this is given by $c^{-1}(V)(1-w_{B})$. Call that the ‘uncontested win threshold.’ If $\theta _{A}<\theta _{B}$, party *A* wants to make sure that the constituencies to their left (their ‘home turf,’ to borrow a phrase from Eyster and Kittsteiner (2007)) have headstarts large enough that elections become uncontested wins. If the uncontested win threshold is high, this may mean shifting the platform left and so widening platform divergence. Alternatively, reducing the whip rate may increase the headstart in those home turf constituencies.

Party *A* makes a tradeoff when moving its platform closer to *B*. It increases *A*’s headstart in the constituencies between their two platforms, and so they do better there. However, it reduces the headstart in the constituencies on the other side of their platform (so, to the left of the platform, for a left-wing party), which may put the home turf at risk. Importantly, I find that the left (right) party chooses to set its platform and whip rate such that all constituencies to its left (right) are *just* uncontested wins—in other words, the headstart in those constituencies is equal to the uncontested win threshold.^8^ This is intuitive: were the left party to move further left, and thereby increase its headstart in those constituencies, they would still be uncontested wins and so no further utility would be gained there—but they would do worse in all the constituencies to their right. But if the left party moved to the right, then they would have no uncontested wins, a key source of party utility.

Figures 2 and 3 show that as $\sigma$ increases, the whip rate increases and platform divergence declines. When uncertainty about candidates is great, a higher whip rate, which reduces the importance of candidates, is more likely to be preferred by a voter. When parties increase their whip rates to attract more votes, the uncontested win threshold falls. Hence, parties can afford to move closer to one another, to capture constituencies between their platforms, without sacrificing their home turfs. Thus, platform divergence falls. Once $\sigma$ has risen beyond a certain point, the costs of voter uncertainty become so large that the only equilibrium is the Downsian one. On the other end, if $\sigma$ is too low, there generally are no pure Nash equilibria. Sharper voter polarization tends to shift the degree of platform differentiation up and the whip rate down, as well as allowing divergent platform equilibria at higher levels of $\sigma$. That is because more polarization incentivizes a lower whip rate (as discussed momentarily), and a higher $\sigma$ offsets that incentive.

**Fig. 4:**
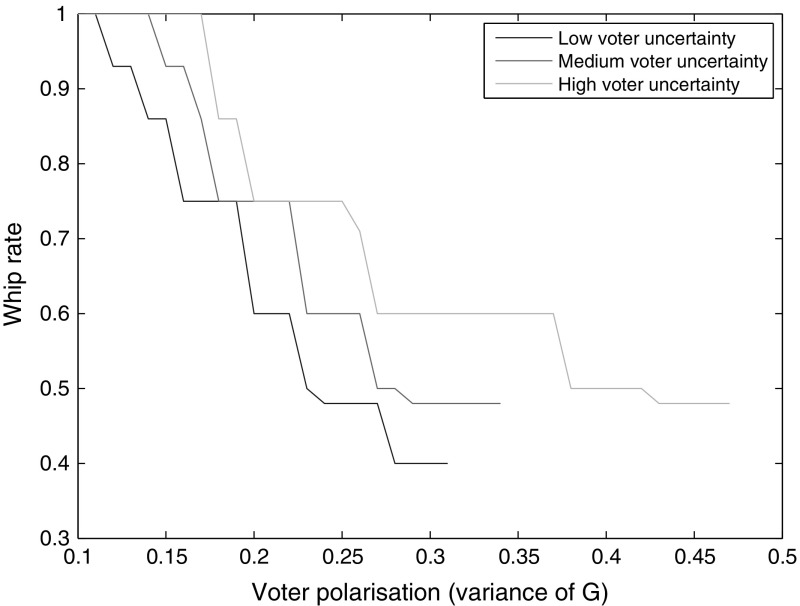
Equilibrium whip rate as a function of voter polarization (variance of *G*), for different values of $\sigma$ (0.20, 0.22, 0.24)

**Fig. 5:**
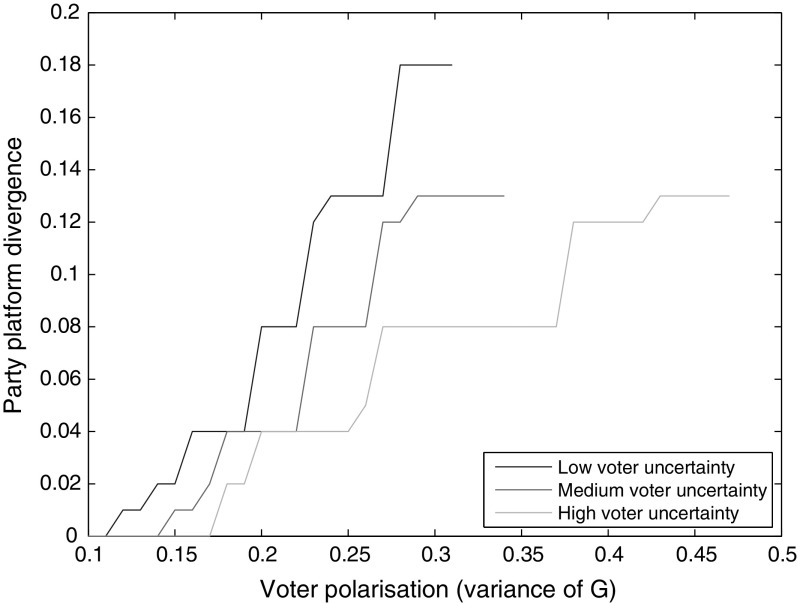
Equilibrium platform divergence as a function of voter polarization (variance of *G*), for different values of $\sigma$ (0.20, 0.22, 0.24)

Figures 4 and 5 show that greater voter polarization is associated with greater platform divergence and a lower whip rate. As voters become more polarized, it is more important for parties to appeal to extreme voters, as there are more of them. That can be done in a straightforward way by making the party platform more extreme, thereby increasing platform divergence. Another option is to reduce the whip rate. A high whip rate is likely to garner votes from voters close to the party platform, but be less successful with voters located further away. When polarization is high and voters are more spread out, a lower whip rate will be preferred by parties in order to appeal to those more extreme constituencies.

How well does the model fit stylized facts about polarization, platform divergence and whip rates? The USA, being a rare example of an almost purely two-party system, serves as the natural ground to answer that question. Some evidence exists that geographic polarization—the sorting of voters into left and right states, and the type of polarization relevant for this study—has increased since the 1980s (see Sussell and Thomson (2015), though for a more skeptical take see Fiorina and Abrams (2008)). Consistent with the model presented herein, party platform divergence generally also is thought to have increased over the same period (for example, see Barber and McCarty (2013) and Merrill et al. (2014)). Trends in the whip rate are rather harder to measure, with researchers tending to focus on more easily measurable party unity scores. We can instead make a comparison to the UK, which serves as a paradimic example of highly whipped parties. Adams et al. (2012) argue that since the 1980s, the British public has become modestly less polarized on the issues, in opposition to the trends seen in the USA. That conclusion coheres with the model’s prediction of less polarized electorates resulting in higher whip rates. These observations are only minimally suggestive, and of course many other factors, not only the myriad of institutional ones, but even other variables that this model captures (such as voter uncertainty) affect platform divergence and whip rates. A full empirical study analyzing these issues would be most welcome.

### Legislator behavior in free votes

It might seem to be an unattractive aspect of the model that the two candidates in any constituency always choose the same platform; even if their *parties* diverge, they do not. However, since candidates aim their platforms at the median voter in their constituencies, and candidates from the left-wing party are more likely to win in left-wing constituencies, the model does imply that *legislators* from a left-wing party are more left wing than those from a right wing party. To illustrate this point, I calculate the average platform of legislators for each party for a particular parameterization.

**Fig. 6:**
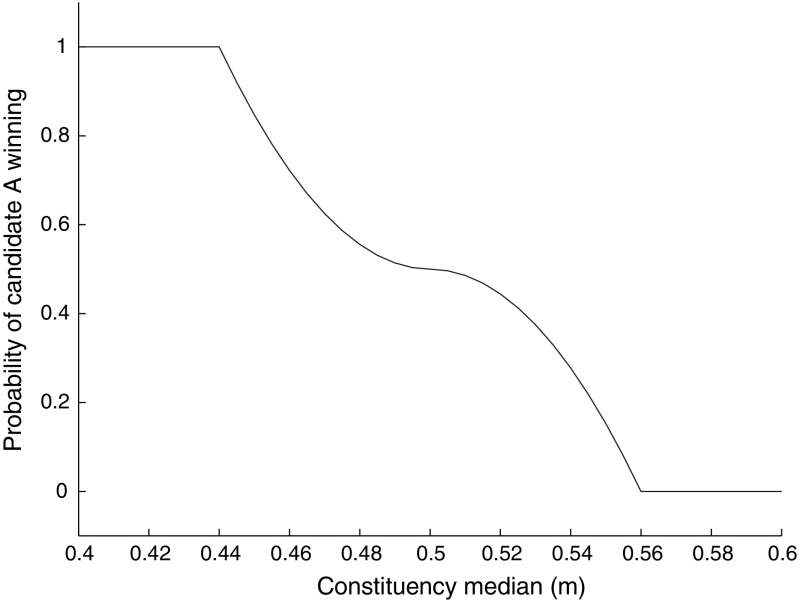
Probability that candidate *a* wins their constituency

With $\sigma =0.2$ and a variance of *G* of 0.25, the simulation finds an equilibrium at $w_{A}=w_{B}=0.5$, $\theta _{A}=0.44$ and $\theta _{B}=0.56$. By the definition of an uncontested win, *a* gets uncontested wins in constituencies where the median $m\le 0.44$; similarly, *b* gets uncontested wins where $m\ge 0.56$. Candidate *a* is upper and lower advantaged in constituencies where $m\in (0.44,0.5)$, and *b* where $m\in (0.5,0.56)$. Applying Theorem  2, we can find the probability that *a* wins in each of these constituencies, as depicted in Fig. 6. The further left is the constituency median, the more likely it is to be won by the left-wing party.

For this parameterization, the average platform of legislators aligned with *A* is 0.32, and for *B* it is 0.68. Hence, party *A*’s legislators are substantially more left than party *B*’s, and indeed in this case the average legislator platform in a given party is more extreme than the party platform.

Therefore, although the model implies that two candidates in any given constituency adopt the same platform, it does not imply that legislators from the left-wing party have the same ideological views as legislators from the right wing party. Instead, the model predicts the observed reality, namely that there is a covariance between a party’s platform and the behavior of its legislators in free votes.

### How do the results change if parties want to win seats?

Until now we have been assuming that parties aim to maximize the sum of their candidates’ utilities. Of course, in fact, parties typically want to increase the number of seats that they hold in the legislature, perhaps because they are office-motivated or because they are policy-motivated. Research in spatial voting focused on models with many constituencies has mostly modeled parties as gaining more utility the more seats they hold (all of the papers discussed in Sect. 2 which model party behavior have this feature, with the exception of Eyster and Kittsteiner (2007) which assumes that party utility is the sum of candidates’ utilities, as we have done up to this point). An alternative—and probably more realistic—arrangement is to assume that parties gain utility from winning a simple majority of legislative seats. This assumption would reflect the benefits that parties enjoy from gaining a majority, such as the executive office and associated patronage, and the chance to pass a policy platform. For this model, I use the former assumption, since the latter introduces awkward discontinuities in the utility functions of parties. However, further research that incorporates the benefits of holding a majority would be most welcome. I model party *A*’s utility as:

$$\begin{aligned} U_{A}=&(1-\delta )\left( \int _{0}^{1}v_{a,m}g(m)\,{\text {d}}m\right) +\delta \left( \int _{0}^{1}\varOmega P(m)g(m)\,{\text {d}}m\right) \end{aligned}$$

Here, *P*(*m*) is the proportion of seats *A* won with a constituency median of *m*, $\delta \in [0,1]$ is a weighting parameter, and $\varOmega$ is the value the party receives from winning a seat. Recall also that $v_{a,m}$ is the utility of candidate *a* in constituency *m*. This equation therefore makes party utility a weighted average of the sum of candidate utilities and the proportion of seats won.

**Fig. 7:**
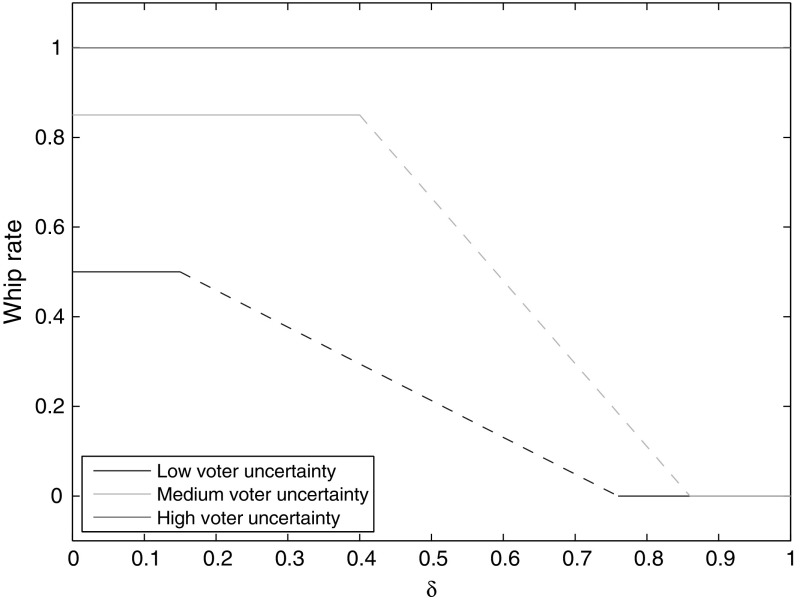
Equilibrium whip rate as a function of $\delta$, for different values of $\sigma$ (0.20, 0.24, 0.35). *Lines* are *dashed* where no pure Nash is available

I simulate the game under this new assumption, setting the variance of *G* to 0.25. I find that the earlier results are robust to this change in assumption so long as $\delta$ is not too high. The equilibrium whip rate and platform divergence are unchanged. This can be seen in Fig. 7, which shows the equilibrium whip rate against $\delta$ and for different levels of $\sigma$.^9^ In order to ease exposition, $\varOmega$ is set equal to the equilibrium utility that parties receive when $\delta =0$ (call this $U^{*}$).

The reason that the results are unchanged is that the aggregate candidate utility achieved at equilibrium ($U^{*}$) is quite high relative to deviations, and so parties will not sacrifice that high utility unless they have a strong focus on seat maximization for its own sake. As discussed above, *A* sets its platform and whip rate such that the constituencies to its left are equal to the uncontested win threshold. Were *A* to move toward the center, in order to capture more seats, all of the constituencies to the left would no longer be uncontested wins. This means that parties have a lot to lose from sacrificing their home turfs, and so are prepared to do so only if they put a high value on winning seats alone. The divergent platform equilibrium ‘survives’ with a higher $\delta$ when voter uncertainty is high, because voters are more averse to a low whip rate.

For some larger values of $\delta$, no pure Nash equilibrium exists (represented in Fig. 7 with dashed lines). That result seems to be because one party can deviate profitably from the divergent platform equilibrium to a strategy where they do not whip any issues, to which the other can in turn best reply with a Downsian strategy (that is, whipping every issue and setting its platform equal to the median constituency). Preliminary testing suggests that in these areas where no pure Nash equilibrium emerges, there may be mixed Nash equilibria wherein players mix between their normal divergent platform strategy and a zero whip rate strategy. A fuller analysis here would be interesting.

When $\delta$ is high, I find that parties will move to one of two strategies. If voter uncertainty is low, they set their whip rates to zero (and so platform is irrelevant), and if voter uncertainty is high, they play a Downsian strategy. In the former case, not whipping ensures that the party platform does not cause any disutility for voters, allowing candidates alone to appeal to their constituencies. In the latter case, a Downsian strategy ensures that voter uncertainty does not cause any disutility for voters.

These results lend some further credence to the earlier results on the responses of whip rates and platform divergence to changes in voter uncertainty and polarization: even when parties are partly seat-seeking for it own sake, they continue to whip less than fully and diverge their platforms in order to deliver uncontested wins for some of their candidates. However, more single-minded seat-seeking parties will choose to either tightly whip their legislators or leave them free to vote as they please, according to whether their incentive to relieve voters of uncertainty or to not put them off with an unappealing platform is stronger.

## Conclusion

I introduced a new choice variable for parties into the spatial voting model with many heterogeneous constituencies: whipping. Under many parameterizations, parties choose to differentiate their platforms from one another, and partially whip their legislators, but also afford them some freedom. Fundamentally, they diverge and whip in order to carve out a ‘home turf,’ wherein their candidates can win elections without having to advertise. In this respect, whipping is a way of cheaply providing greater certainty to an imperfectly informed electorate. The ‘uncontested wins’ that parties aim for are parallel to the real-world phenomenon of ‘safe seats,’ where candidates can win elections with near certainty, without much campaigning. However, constituencies also exist that are contested and incumbents may lose—mirroring the fact that not all seats are safe. This contrasts to, for example, the model in Snyder (1994), where although parties (slightly) differentiate their platforms, every incumbent legislator is reelected with certainty. The uncertainty regarding the winner of contested seats likewise is endogenous to my model, as opposed to resulting from exogenous shocks such as in Ansolabehere et al. (2012). The result of some whipping, but not complete party power, also reflects an empirical reality seen across countries, suggesting that while parties are influential, legislators also are able to vote as they please in at least some votes.

Furthermore, although the model does imply that candidates in a given constituency adopt the same platforms, it predicts that legislators from a left-wing party tend to be more left wing than legislators from a right wing party, even when they aren’t being whipped. Again, this is endogenous to the model rather than resulting from imposed ideological motivations in candidate utility functions.

In these respects, a relatively parsimonious model is able to generate a reasonably realistic picture of electoral politics. Hence, I tentatively suggest that whipping, thus far ignored in the literature, should be further explored in the spatial modeling of elections.

Platform divergence and whip rates are both sensitive to the degree of voter polarization and voter uncertainty about candidates. Most interestingly, parties respond to greater polarization among voters by not just becoming more polarized in their own platforms, but also by reducing their whip rates and giving legislators more freedom. By reducing the importance of the party brand, parties allow candidates to appeal more directly to their constituency electorates. A empirical test of this hypothesis would be a valuable extension.

These results also turn out to be relatively robust to a change in assumptions about party motivations. If parties partly seek to win as many seats as possible, their incentive to deliver uncontested wins for many of their candidates remains sufficiently strong to ensure that they continue to whip only some issues and differentiate their platforms to some degree. The results do change, however, for more purely seat-seeking parties; such a party will either whip all issues or none, in order to maximize seat wins.

The results presented here are for a two-party electoral system with single-member legislative districts, such as that for the US Congress and to some extent the UK Parliament. Further extensions would be required to draw conclusions about other sorts of electoral systems. But perhaps the most exciting addition to the model would be to open the ‘black box’ of whipping. I assumed that parties could successfully whip all the issues they choose. However, in reality, of course, legislators sometimes rebel against the whip. A model could be designed that includes the incentives that legislators face regarding obeying or disobeying the whip, potentially when the legislator rebels if she fears that toeing the party line could result in a backlash from her constituents at the next election. This would then provide further incentives for the party to carefully choose its whip rate and manifesto, in order to successfully pass legislation.

## Appendix

### **Proposition 1**

*Parties’ and candidates’ optimal platform choice always includes choosing the same position for all issues rather than different positions for different issues; that is, if party* *A* *whips issues* $1,\ldots ,{\bar{r}}$, *they will find that setting* $\theta _{A,r}=\theta _{A}\forall r\in \{1,\ldots ,\bar{r}\}$, for some *platform* $\theta _{A}$ *will be optimal; similarly, setting* $\theta _{a,i,s}=\theta _{a,i}\forall s\in \{\bar{r}+1,\ldots ,R\}$ *for some platform* $\theta _{a,i}$ *will be optimal for* *a*.

For brevity I drop constituency subscripts. I prove that a party or candidate deviating from this strategy will make some voters worse off and no voters better off; since parties and candidates do better, *ceteris paribus*, when they make voters better off, and there are no other strategic incentives to deviate, it is always optimal to play this strategy.

First, define an overall weighted average of the platforms for all whipped issues, $\theta _{A}$:

$$\begin{aligned} \theta _{A}= \frac{\sum _{r=1}^{\bar{r}}\rho _{r}\theta _{A,r}}{\sum _{r=1}^{\bar{r}} \rho _{r}} \end{aligned}$$

Suppose that party *A* sets its whipped policies $\{\theta _{A,1},\ldots ,\theta _{A,\bar{r}}\}=\{\theta ^{\prime }_{A,1},\ldots ,\theta ^{\prime }_{A,\bar{r}}\}=\mathbb {X}$. $\{\theta ^{\prime }_{A,1},\ldots ,\theta ^{\prime }_{A,\bar{r}}\}$ are such that all policies are equal, so $\theta ^{\prime }_{A,1}=\cdots =\theta ^{\prime }_{A,\bar{r}}=\theta ^{\prime }_{A}$. Now consider the utility that voter *j* with an ideological bliss point $y_{j}$ gets from *A*’s whipped policies alone:

$$\begin{aligned} u_{j}(\mathbb {X})= -\sum _{r=1}^{\bar{r}}\rho _{r}|y_{j}-\theta ^{\prime }_{A}| \end{aligned}$$

Suppose that party *A* now changes its platform on issues $\hat{r}+1,\ldots ,\bar{r}$, so $\{\theta _{A,\hat{r}+1},\ldots ,\theta _{A,\bar{r}}\}=\{\theta ^{\prime \prime }_{A,\hat{r}+1},\ldots ,\theta ^{\prime \prime }_{A,\bar{r}}\}$ (and define the new set of platforms as $\mathbb {Y}$) such that its weighted average platform is unchanged, so:

$$\begin{aligned} \theta ^{\prime \prime }_{A}= \theta ^{\prime }_{A}=\frac{\Bigl (\sum _{r=1}^{\hat{r}}\rho _{r} \theta ^{\prime }_{A}\Bigr )+\Bigl (\sum _{r=\hat{r}+1}^{\bar{r}} \rho _{r}\theta ^{\prime \prime }_{A,r}\Bigr )}{\sum _{r=1}^{\bar{r}}\rho _{r}} \end{aligned}$$

This also entails that the changed platforms $\hat{r}+1,\ldots ,\bar{r}$ must have a weighted average equal to the original overall weighted average:

A.1 $$\begin{aligned} \frac{\left( \sum _{r=\hat{r}+1}^{\bar{r}}\rho _{r} \theta ^{\prime \prime }_{A,r}\right) }{\sum _{r=\hat{r}+1}^{\bar{r}}\rho _{r}}=\theta ^{\prime }_{A} \end{aligned}$$

*j*’s utility from *A*’s whipped policies is now:

$$\begin{aligned} u_{j}(\mathbb {Y})= -\left( \left( \sum _{r=1}^{\hat{r}}\rho _{r}|y_{j} -\theta ^{\prime }_{A}|\right) +\left( \sum _{r=\hat{r}+1}^{\bar{r}} \rho _{r}|y_{j}-\theta ^{\prime \prime }_{A,r}|\right) \right) \end{aligned}$$

Assume that $y_{j}>\theta ^{\prime }_{A}$ (a symmetric proof is available if the reverse is true). Assume further, without loss of generality, that $\theta _{A,\hat{r}+1},\ldots ,\theta _{A,\underline{r}}<y_{j}$ and $\theta _{A,\underline{r}+1},\ldots ,\theta _{A,\bar{r}}\ge y_{j}$. The difference in *j*’s utility between these two strategies is:

$$\begin{aligned} u_{j}(\mathbb {X})-u_{j}(\mathbb {Y})= \left( \left( \sum _{r=1}^{\hat{r}} \rho _{r}(y_{j}-\theta ^{\prime }_{A})\right) +\left( \sum _{r=\hat{r}+1}^{\bar{r}}\rho _{r}|y_{j}-\theta ^{\prime \prime }_{A,r}|\right) \right) -\sum _{r=1}^{\bar{r}}\rho _{r}(y_{j}-\theta ^{\prime }_{A}) \end{aligned}$$

Splitting out the middle and right-hand sums, adding and subtracting $\sum _{r=\underline{r}+1}^{\bar{r}}\rho _{r}\theta ^{\prime \prime }_{A,r}$ and simplifying:

$$\begin{aligned}&=\left( \sum _{r=\hat{r}+1}^{\underline{r}}\rho _{r}\left( \theta ^{\prime }_{A} -\theta ^{\prime \prime }_{A,r}\right) \right) +\left( \sum _{r=\underline{r}+1}^{\bar{r}}\rho _{r}\left( \theta ^{\prime \prime }_{A,r} +\theta ^{\prime }_{A}-2y_{j}\right) \right) \\&= \theta ^{\prime }_{A}\left( \sum _{r=\hat{r}+1}^{\bar{r}}\rho _{r}\right) -\left( \sum _{r=\hat{r}+1}^{\bar{r}}\rho _{r}\theta ^{\prime \prime }_{A,r}\right) +\left( \sum _{r=\underline{r}+1}^{\bar{r}}2\rho _{r} \left( \theta ^{\prime \prime }_{A,r}-y_{j}\right) \right) \end{aligned}$$

Substituting a rearranged (A.1) gives:

$$\begin{aligned}&= \theta ^{\prime }_{A}\left( \sum _{r=\hat{r}+1}^{\bar{r}}\rho _{r}\right) -\theta ^{\prime }_{A}\left( \sum _{r=\hat{r}+1}^{\bar{r}}\rho _{r}\right) +\left( \sum _{r=\underline{r}+1}^{\bar{r}}2\rho _{r}\left( \theta ^{\prime \prime }_{A,r}-y_{j}\right) \right) \\&= \sum _{r=\underline{r}+1}^{\bar{r}}2\rho _{r}(\theta ^{\prime \prime }_{A,r}-y_{j})\ge 0 \end{aligned}$$

This inequality will be strict for voters *j* for whom $\theta _{A,r}>y_{j}\exists \theta _{A,r}\in \{\theta ^{\prime \prime }_{A,\hat{r}+1},\ldots ,\theta ^{\prime \prime }_{A,\bar{r}}\}$.

Hence, at least some voters will be made worse off by this change and none made better off. This is true for any overall weighted platform average $\theta _{A}$. So, for any set of platforms $\{\theta _{A,1},\ldots ,\theta _{A,\bar{r}}\}$, it is better for party *A* to achieve that same overall weighted platform average with the same platform for all issues. Similar arguments apply for party *B* and candidates *a* and *b*.

### **Proposition 2**

*If* $\sigma <c^{-1}(V)$ *, there is not generally a Nash equilibrium*

This proof depends upon Lemmas 1 and 2 and the definitions in the text.

Suppose $\sigma <c^{-1}(V)$ and $w_{A}<1$, $w_{B}<1$. Suppose also that the election is contested and that *a* is both upper and lower advantaged. As stated in Sect. 3, the maximum $e_{i}$ allowed is $e_{i}=\sigma$. As shown in Lemma 1, all $e_{a}>\frac{\sigma (1-w_{B})-H}{1-w_{A}}$ are strictly dominated. Lemma 2 shows that there is no mass on any $e_{i}>0$, for $i\in \{a,b\}$.

We can see that *b*, the lower disadvantaged candidate, will not play $e_{b}=0$. If she did, she would lose for sure; but if she plays $e_{b}=\sigma$, she wins for sure (as *a* does not play above $\frac{\sigma (1-w_{B})-H}{1-w_{A}}$), and gets positive utility (since, by assumption, $\sigma <c^{-1}(V)$). *b* also will not play $e_{b}\in (0,\frac{H}{1-w_{B}})$, for the same reasons as stated in the proof of Lemma 1. Similar reasoning shows that *a* will therefore not play $e_{a}=0$, as there she would lose for sure. Since, by Lemma 2, the only possible mass points are at $e_{i}=0$, this shows that there are no mass points. Both candidates must therefore mix over some support.

Now, take the lowest $e_{b}$ that *b* plays; call it *x*. Define *y* such that:

A.2 $$\begin{aligned} {\mathbb {E}}[u_{m,b}(e_{b}=x)]={\mathbb {E}}[u_{m,a}(e_{a}=y)] \end{aligned}$$

So *y* is such that if *a* plays $e_{a}=y$ and *b* plays $e_{b}=x$, it will be a tie since the median voter is indifferent between *a* and *b*. Since *x* is the lowest that *b* will play, *a* will be unwilling to play beneath *y*, since if she does she will lose for sure, and thus get non-positive utility; but she could play $e_{a}=\frac{\sigma (1-w_{B})-H}{1-w_{A}}$, win for sure and get positive utility. The same is true the other way around; if the lowest *a* plays is *y*, *b* will be unwilling to play beneath *x*. Hence, the lowest $e_{i}$ in each candidate’s support will be such that (A.2) holds. But since there are no mass points, if either candidate plays at the bottom of their support (i.e., if either play *x* or *y*), they will lose for sure and receive non-positive utility.

So, we have a contradiction: In equilibrium, both candidates must mix over some support and so are indifferent between playing any $e_{i}$ in that support. But, for any possible support, at least one candidate will have a lower expected utility if they play at the bottom of their support than if they play at $\sigma$ (for *b*, the upper disadvantaged candidate) or $\frac{\sigma (1-w_{B})-H}{1-w_{A}}$ (for *a*, the upper advantaged candidate). Thus, it is not possible for both candidates to mix over some support in equilibrium. So, there is no Nash equilibrium.

### **Lemma 1**

1. *If* *a* *is lower advantaged, then for* *b*, *all* $e_{b}\in (0,\frac{H}{1-w_{B}})$ *are strictly dominated.*
2. *If* *a* *is upper advantaged (or neither candidate is upper advantaged), then for* *a*, *all* $e_{a}>\frac{c^{-1}(V)(1-w_{B})-H}{1-w_{A}}$ *are strictly dominated.*

1. Suppose that *a* is lower advantaged. If $e_{a}=0$, then *a* wins if:
  $$\begin{aligned} H>&e_{b}(1-w_{B}) \end{aligned}$$
  This will hold for all $e_{b}\in \Bigl [0,\frac{H}{1-w_{B}}\Bigr )$. Hence, if *b* plays $e_{b}\in \Bigl (0,\frac{H}{1-w_{B}}\Bigr )$, she will pay a positive cost but will never win, and so any $e_{b}$ in that set is strictly dominated by $e_{b}=0$.
2. If *b* is not upper advantaged (so, *a* is upper advantaged or neither *a* nor *b* are upper advantaged), then, since *b* will never play above $c^{-1}(V)$, *a* will win for sure so long as:
  $$\begin{aligned} H>&c^{-1}(V)(1-w_{B})-e_{a}(1-w_{A}) \end{aligned}$$
  This holds so long as $e_{a}>\frac{c^{-1}(V)(1-w_{B})-H}{1-w_{A}}$. If *a* is playing $e_{a}=\frac{c^{-1}(V)(1-w_{B})-H}{1-w_{A}}+\epsilon$, with any $\epsilon >0$, she can reduce $e_{a}$ marginally and still win for sure but with a lower cost. So all $e_{a}>\frac{c^{-1}(V)(1-w_{B})-H}{1-w_{A}}$ are strictly dominated by a slightly lower $e_{a}$.

### **Lemma 2**

*Neither player puts a mass point on any* $e_{i}$ *other than zero*

Firstly, suppose the election is uncontested in *a*’s favor. Then, any $e_{b}>0$ is strictly dominated by $e_{b}=0$; by the definition of an uncontested election, the $e_{b}$ required to give *b* a chance at winning would give her a negative utility if she did win. So *b* plays $e_{b}=0$, to which the optimal reply is $e_{a}=0.$ So both players put mass at zero and nowhere else. The same is true when the election is uncontested in *b*’s favor.

Secondly, suppose the election is contested, and that *a* is upper advantaged. I prove that *a* will not put mass anywhere other than zero in three parts. Firstly, *a* will not put mass $\in (0,\bar{e_{a}})$. Suppose *a* did put mass at $e_{a}=x\in (0,\bar{e_{a}})$, and define *y* such that:

$$\begin{aligned} {\mathbb {E}}[u_{m,a}(e_{a}=x)]=&\,{\mathbb {E}}[u_{m,b}(e_{b}=y)] \end{aligned}$$

So *y* is such that if *b* plays $e_{b}=y$ and *a* plays $e_{a}=x$, the median voter is indifferent between *a* and *b*.

Then, there is a set $[y-\epsilon ,y]$, for some $\epsilon >0$ where *b* will not play, as if she plays just above *y* instead she will increase the probability of winning discretely, but only continuously increase costs. However, if *b* does that, *a* would move the mass point down slightly from *x*, as it would reduce her costs but not change the probability of winning. So there cannot be a mass point at *x*. Secondly, there is no equilibrium where *a* is upper advantaged and places mass at $e_{a}=\bar{e_{a}}$. Then, *b* would do better to not play $e_{b}\in (c^{-1}(V)-\epsilon ,c^{-1}(V))$, as moving to $e_{b}=c^{-1}(V)$ discretely increases the probability of winning (by getting a tie). If *b* did indeed play $e_{b}=c^{-1}(V)$, then *a* could discretely increase the probability of winning by moving her mass point up marginally from $e_{a}=\bar{e_{a}}$. If *b* did not play $e_{b}\in (c^{-1}(V)-\epsilon ,c^{-1}(V)]$, then *a* could move her mass point down marginally, reducing cost but not changing the chance of winning. Thirdly, *a* will not put mass at $e_{a}>\bar{e_{a}}$, since any $e_{a}>\bar{e_{a}}$ is strictly dominated (see Lemma 1). Thus, there is no equilibrium where *a* places a mass point at $e_{a}>0$.

Similarly, *b* (the upper disadvantaged candidate) will not put mass in $e_{b}\in (\underline{e}_{b},\bar{e_{b}})$ in equilibrium for similar reasons to above. Also, there is no equilibrium with *b* putting mass at $e_{b}=\underline{e}_{b}$. If *a* puts mass at $e_{a}=0$, then *b* will not want to play at $\underline{e}_{b}$, as if she plays slightly above she will discretely increase the probability of winning. If *a* does not put mass at $e_{a}=0$, then *b* still won’t want to play at $\underline{e}_{b}$ since she will lose for sure, but will pay a positive cost. Lemma 1 also shows that *b* will not put mass at $e_{b}\in (0,\underline{e}_{b})$. Hence, neither player will put mass at any point other than zero.

### **Lemma 3**

*In contested elections:*

1. *If a candidate with lower advantage has a hole in her strategy between* *y* *and* *z* (*with* $y<z$), *then she has a hole between* *y* and $\bar{e}$.
2. *If a candidate without lower advantage has a hole between* *y* *and* *z* (*with* $y<z$), *then she has a hole between* *y* *and* $\bar{e}$, *unless* $0<y<\underline{e}$.

1. Suppose that *a* is lower advantaged and has a hole between *p* and *q* but not between *q* and $\bar{e_{a}}$,where $0<p<q<\bar{e_{a}}$. Define *r* and *s* such that:
  $$\begin{aligned} {\mathbb {E}}[u_{m,a}(e_{a}=p)]=&\,{\mathbb {E}}[u_{m,b}(e_{b}=r)]\\ {\mathbb {E}}[u_{m,a}(e_{a}=q)]=&\,{\mathbb {E}}[u_{m,b}(e_{b}=s)] \end{aligned}$$
  By Lemma 2, *a* will not put mass on *p* or *q*, and nor will *b* put mass on *r* or *s*. *b* will be unwilling to play $e_{b}\in (r,s)$, since her probability of winning is the same as when $e_{b}=r$, but her cost higher. So *b* has a hole between *r* and *s*. However, now *a* will be unwilling to play $e_{a}\in [q,q+\epsilon )$ for some small $\epsilon >0$—since she could play somewhere in (*p*, *q*) instead, and discretely reduce costs but only continuously reduce the probability of winning. But this contradicts our assumption that there is no hole between *q* and $\bar{e_{a}}$. Thus, if there is a hole for *a* between *p* and *q*, there must be one between *p* and $\bar{e_{a}}.$
2. A very similar argument to above proves this element of the lemma. The restriction regarding $y<\underline{e}$ is needed because the candidate without lower advantage *will *have a hole where her level of advertising is strictly dominated, even though she does not have a hole above $\underline{e}$—see Lemma 1.

### **Lemma 4**

*Suppose at least one candidate is either lower or upper advantaged. Then: *

1. *If one candidate does not have lower advantage and the other does not have upper advantage, the first does not put mass on zero but the second does.*
2. *If one candidate is upper and lower advantaged, both candidates put mass on zero*.

The condition at the start of this lemma—that at least one candidate has some advantage—is to cover the special case where both candidates are neither lower nor upper advantaged. In that case it is easy to show that no candidate puts mass anywhere^10^.

1. Let *a* be upper advantaged and *b* be lower advantaged^11^.

Then, by Lemma 1, their set of undominated strategies are:

$$\begin{aligned} e_{a}\in&\,\left\{ 0,\left[ \underline{e}_{a},\frac{c^{-1}(V)(1-w_{B})-H}{1-w_{A}}\right] \right\} \\ e_{b}\in\,&[0,c^{-1}(V)] \end{aligned}$$

Suppose that *b* does not place mass at zero. By Lemma 2, this means that she does not place a mass point anywhere. Then, *a* will not want to play $e_{a}\in [\underline{e}_{a},\underline{e}_{a}+\epsilon ]$, for some arbitrarily small $\epsilon >0$—because if a played in that range she could reduce costs discretely to $e_{a}=0$, and only continuously reduce the probability of winning. But this means that the lower disadvantaged candidate has a hole in her strategy and the hole is above $\underline{e}_{a}$. By Lemma 3, this entails that she has a hole from $\underline{e}_{a}$ to $\bar{e_{a}}$, and since we already know from Lemma 1 that she doesn’t play $e_{a}\in (0,\underline{e}_{a})$, that entails that she plays $e_{a}=0$ for sure. The best reply to this is $e_{b}=0.$ But this means that that *b* *is *placing a mass point at zero! Therefore, the lower advantaged candidate places mass at zero.

It straightforwardly follows that the upper advantaged but lower disadvantaged candidate—in this case *a*—will not place mass on zero. If she plays zero, she loses for sure, by the definition of lower disadvantage, and so would receive a utility of zero. However, if she plays $\bar{e_{a}}=\frac{c^{-1}(V)(1-w_{B})-H}{1-w_{A}}$, she will win for sure and, since $\bar{e_{a}}<c^{-1}(V)$ for the upper advantaged candidate, she will receive positive utility. So, she will be unwilling to play zero.

2. Let *a* be upper and lower advantaged. Then, by Lemma 1, their set of undominated strategies are:
  $$\begin{aligned} e_{a}\in&\,\left[ 0,\frac{c^{-1}(V)(1-w_{B})-H}{1-w_{A}}\right] \\ e_{b}\in&\,\{0,[\underline{e}_{b},c^{-1}(V)]\} \end{aligned}$$

The proof that in this scenario *a* will put mass on zero is the same as the proof in part (1) that *b* will put mass on zero. To prove that here *b* will also put mass on zero: Suppose that she did not, and so always played at least $\underline{e}_{b}$. Then, when *a* plays zero, *a* will lose for sure and so will receive zero utility. However, if *a* plays $\bar{e_{a}}=\frac{c^{-1}(V)(1-w_{B})-H}{1-w_{A}}$, she will win for sure and receive positive utility. This means that *a* will not play zero—which contradicts our above claim that she will put a mass point on zero. So it must be the case that *b* puts a mass point on zero.

### **Lemma 5**

*If the election is contested, both candidates have* $\bar{e_{i}}$ *in their support*

Suppose that *a* is upper advantaged. The fact that *b* gets zero utility in equilibrium (from Lemma 4) implies that *a* must have $e_{a}=\bar{e_{a}}=\frac{c^{-1}(V)(1-w_{B})-H}{1-w_{A}}$ in her support; if she did not, then *b* could play near $e_{b}=\bar{e_{b}}=c^{-1}(V)$, win for sure and collect positive utility. This in turn implies that *b* must have $e_{b}=c^{-1}(V)$ in her support; else, *a* would not want to play $e_{a}=\frac{c^{-1}(V)(1-w_{B})-H}{1-w_{A}}$, as she could play slightly less and still win for sure.

### **Theorem 1**

E1.: *When the election is uncontested, neither candidate has any incentive to spend on advertising. If the election is uncontested in* *a*’*s favor, then the only way that* *b* *could hope to win would be to spend* $e_{b}\ge c^{-1}(V)$ *on advertising. However, this clearly would give negative utility in expectation.* *Any* $0<e_{b}<c^{-1}(V)$ *would result in a certain loss for* *b*, *and thus negative utility. Hence,* $e_{b}=0$ *is the strictly dominant strategy. Since* *b* *plays* $e_{b}=0$, *a* *will win for sure regardless of what* $e_{a}$ *is. Since her utility is* *decreasing in* $e_{a}$, *she sets* $e_{a}=0$.
E2.: *The case referred to here is slightly awkwardly stated to take account of cases where, for example, one candidate is upper advantaged but neither are lower advantaged. These special cases are all covered by the stated strategies, but to save space I just prove E2 in the generic case where* *a* *is upper advantaged and* *b* *is lower advantaged. Since* ${\mathbb {E}}[v_{b}]=0$ *(as shown in the discussion of Lemma* 4), *we have to find what strategy* *a* *must play to give* ${\mathbb {E}}[v_{b}]=0$ *for all* $e_{b}$ *in the support* $(0,c^{-1}(V))$: $$\begin{aligned} {\mathbb {E}}[v_{b}|e_{b}=x:0\le x\le c^{-1}(V)]=&\,\Pr (H<x(1-w_{B})-e_{a}(1-w_{A}))V-c(x)\\ =&\,F_{a}^{*}\left( \frac{x(1-w_{B})-H}{1-w_{A}}\right) V-c(x)=0\\ \Rightarrow&\,F_{a}^{*}\left( \frac{x(1-w_{B})-H}{1-w_{A}}\right) =\frac{c(x)}{V}\\ \Rightarrow&\,F_{a}^{*}(x)=\frac{1}{V}c\left( \frac{x(1-w_{A})+H}{1-w_{B}}\right) \end{aligned}$$

*To find* $F_{b}$, *we use the fact that when* *a* *plays* $e_{a}=\frac{c^{-1}(V)(1-w_{B})-H}{1-w_{A}}$ *she wins for sure, and so her utility across her support must be:*

$$\begin{aligned} {\mathbb {E}}[v_{a}]=&V-c\left( \frac{c^{-1}(V)(1-w_{B})-H}{1-w_{A}}\right) \end{aligned}$$

*Hence:*

$$\begin{aligned} {\mathbb {E}}\left[ v_{a}|e_{a}= \frac{-H}{1-w_{A}}\le x\le \frac{c^{-1}(V)(1-w_{B})-H}{1-w_{A}}\right] =&\Pr (H>e_{b}(1-w_{B})-x(1-w_{A}))V-c(x)\\ \Rightarrow&F_{b}^{*}\left( \frac{H+x(1-w_{A})}{1-w_{B}}\right) V-c(x)= V-c\left( \frac{c^{-1}(V)(1-w_{B})-H}{1-w_{A}}\right) \\ \Rightarrow&F_{b}^{*}\left( \frac{H+x(1-w_{A})}{1-w_{B}}\right) = 1-\frac{1}{V}(c\left( \frac{c^{-1}(V)(1-w_{B})-H}{1-w_{A}}\right) -c(x))\\ \Rightarrow&F_{b}^{*}(x)= 1-\frac{1}{V}\left( c\left( \frac{c^{-1}(V)(1-w_{B})-H}{1-w_{A}}\right) -c\left( \frac{x(1-w_{B})-H}{1-w_{A}}\right) \right) \end{aligned}$$

*Finally, since we know the support that* *a* *and* *b* *play, the equilibrium strategies follow.*

*Strictly speaking we need a further assumption:*

*If* ${\mathbb {E}}[u_{j}(a)]={\mathbb {E}}[u_{j}(b)]$, *and either* $w_{A}=1$ *or* $w_{B}=1$, *j* *will vote for the candidate with the lower whip rate, unless both whip rates are* 1, *in which case she votes for each with probability one-half.*

*This could be part of a stronger assumption that in the case of a tie, the voter will always choose the party with the lower whip rate, even if* $w_{A}\ne 1$ *and* $w_{B}\ne 1$; *though this stronger assumption will not make any difference to the equilibrium. We could justify the assumption on the grounds that voters fear that overly powerful parties could have negative consequences for democracy, but that this concern comes second in a lexicographic preference ordering. *

*Without this assumption, there is a case where there is no Nash equilibrium. This is when one party has their whip rate at *1, *the candidate of the other party is upper advantaged, and the election is contested. Suppose that* $w_{B}=1$, *and* *a* is *upper advantaged. Since* $w_{B}=1$, *it is obvious that* $e_{b}=0$ *is the optimal strategy, as* $e_{b}$ *has no impact on* *b*’s *chance of winning.* *Because the election is contested, it must be that* $H<c^{-1}(V)(1-w_{B})=0$; *so* *b* *is lower advantaged. As there is no uncertainty about* *b*’*s action,* *a* *will win outright if:*

$$\begin{aligned} H>&e_{b}(1-w_{B})-e_{a}(1-w_{A})=-e_{a}(1-w_{A})\\ \Rightarrow&\frac{-H}{1-w_{A}}< e_{a}\\ \Rightarrow&\frac{-H}{1-w_{A}}+\epsilon = e_{a} \end{aligned}$$

*For any arbitrarily small* $\epsilon >0$. *But this means that there is no Nash equilibrium, because for any* $e_{a}>\frac{-H}{1-w_{A}}$ *that* *a* *chooses, she could have chosen a slightly lower one, still won, and had a higher payoff. However, if we make the assumption above, then* *a* *can set* $e_{a}=\frac{-H}{1-w_{A}}$ *and win with certainty, making it the unique best response; and so giving us the Nash equilibrium.*

E3.: *The proof of this part of the theorem is similar to E2 as equilibrium utility for both players is the same as in E2.* $F_{a}^{*}$ *is the same as in E2, except with a different support, following Proposition* 2. *For* $F_{b}^{*}$, *we have to take account of the fact that there is now a gap in the support, resulting in the second case.* □

### **Theorem 2**

*Equilibrium E1 is obvious.*

*For E2: Recall that* *a* *wins when* $H>e_{b}(1-w_{B})-e_{a}(1-w_{A})$. *Hence:*

$$\begin{aligned} \Pr (a\text { wins}|e_{a}=x)=&\,\Pr (H>e_{b}(1-w_{B})-x(1-w_{A}))\\ =&\,F_{b}^{*}\biggl (\frac{H+x(1-w_{A})}{1-w_{B}}\biggr )\\ =&\,1-\frac{1}{V}\Bigr (c(\gamma )-c(x)\Bigl ) \end{aligned}$$

*We can then calculate the joint probability of* *a* *winning and* *a* *playing* $e_{a}=x$, *where* $\gamma \ge x\ge \frac{-H}{1-w_{A}}$. *The latter is* *taken by differentiating* $F_{a}^{*}$:

$$\begin{aligned} \Pr (a\text { wins}\wedge e_{a}=x)=&\,\Pr (a\text { wins}|e_{a}=x)\cdot \Pr (e_{a}=x)\\ =&\,\biggl [1-\frac{1}{V}\Bigr (c(\gamma )-c(x)\Bigl )\biggr ]\cdot \frac{1}{V}c^{\prime }\biggl (\frac{x(1-w_{A})+H}{1-w_{B}}\biggr )\biggl (\frac{1-w_{A}}{1-w_{B}}\biggr ) \end{aligned}$$

*Finally, we can use the law of total probability by integrating over the values of* $e_{a}$ *that* *a* *mixes over:*

A.3 $$\begin{aligned} \Pr (a\text { wins})=\int _{\frac{-H}{1-w_{A}}}^{\gamma }\Biggl [1-\frac{1}{V}\biggl (c(\gamma )-c(x)\biggr )\Biggr ]\frac{1}{V}c^{\prime }\biggl (\frac{x(1-w_{A})+H}{1-w_{B}}\biggr )\biggl (\frac{1-w_{A}}{1-w_{B}}\biggr )\,\text {d}x \end{aligned}$$

*For E3, the proof goes in much the same way as for E2. However, there are two differences. Firstly, the range that* *a* *mixes over is* $(0,\gamma )$, *rather than* $(\frac{-H}{1-w_{A}},\gamma )$. *Secondly,* *a* *also puts mass on* 0. *Hence, we need to add the probability that* *a* *puts mass on zero and still wins. This is given by:*

$$\begin{aligned} \Pr (e_{a}=0)\cdot \Pr (e_{b}=0)=&\frac{1}{V}c\biggl (\frac{H}{1-w_{B}}\biggr )\cdot \Biggl (1-\frac{1}{V}\Biggl (c\Biggl (\frac{c^{-1}(V)(1-w_{B})-H)}{1-w_{A}}\Biggr )\Biggr )\Biggr ) \end{aligned}$$

*Changing the bottom limit of the integral in (* A.3 *) to zero and adding on the above term gives the result stated in the third part of the theorem.*□

## Footnotes

1 The slightly awkward statements of ‘not lower advantaged’ and ‘not upper advantaged’ are to cover special cases wherein, for example, one candidate is upper advantaged but neither is lower advantaged. The generic case that this part of the lemma refers to occurs when one candidate is upper advantaged and the other is lower advantaged.

2 Given the condition at the beginning of Lemma 4—that at least one candidate must have at least one sort of advantage—one might expect that a similar sort of qualification needs to be made here. However, the strategies described in E2 also cover the case in which neither candidate has either sort of advantage ($F_{a}^{*}(x)=F_{b}^{*}(x)=\frac{c(x)}{V}$ for $0\le x\le c^{-1}(V)$).

3 For other voter theory models using simulation, see, among others, Kollman et al. (1992), Adams (1999) and Adams et al. (2010).

4 For these simulations, I set $V=0.6$ and $\alpha =0.2$, although other parameterizations give very similar results.

5 For a very small number of parameterizations, an equilibrium exists in which one party plays a whip rate of 0 and the other plays a whip rate near 1 and a platform near $\frac {1}{2}$. Testing suggests that this is an artifact of discretization, with these equilibria becoming increasingly rare the more grid points are added.

6 Usually only one (or zero) equilibria are available for each parameterization; in cases where more than one is available, I record the equilibrium with the larger platform divergence.

7 In these scenarios parties can deviate profitably from a divergent platform strategy by playing a whip rate of zero. That strategy shifts voter attention away from the party and so tends to improve their prospects in extreme constituencies wherein the party’s platform is further from the constituency median. When polarization is sharp, more extreme constituencies exist and so the benefits from such a deviation are larger. When uncertainty is low, the cost of playing a strategy with a zero whip rate is diminished. That is because $\frac{\delta H(m)}{\delta w_{A}\delta \sigma }=1$, so little uncertainty implies (all else equal) a smaller decline in headstart when the whip rate is reduced.

8 When $w_{A}=w_{B}$, if $\theta _{A}<\theta _{B}$ *H* is flat to the left of $\theta _{A}$ and to the right of $\theta _{B}$. So if one constituency to the left of *A* is an uncontested win, so are all to the left of *A*.

9 To facilitate computation, these results have been calculated with a lower level of discretization.
